# A critical opinion-based review of hospital pharmacy compounding with respect to the risk of leachable substances due to the off-label use of plastic primary packaging

**DOI:** 10.1177/20420986251317424

**Published:** 2025-02-13

**Authors:** William Bello, Julian Pezzatti, Camille Stampfli, Laurent Carrez, Serge Rudaz, Farshid Sadeghipour

**Affiliations:** Pharmacy Department, Lausanne University Hospital, Lausanne, Switzerland; Center for Research and Innovation in Clinical Pharmaceutical Sciences, Lausanne University Hospital and University of Lausanne, Lausanne, Switzerland; School of Pharmaceutical Sciences, University of Geneva, Geneva, Switzerland; Institute of Pharmaceutical Sciences of Western Switzerland, University of Geneva, University of Lausanne, Geneva, Switzerland; Pharmacy Department, Lausanne University Hospital, Lausanne, Switzerland; Pharmacy Department, Lausanne University Hospital, Lausanne, Switzerland; Pharmacy Department, Lausanne University Hospital, Lausanne, Switzerland; School of Pharmaceutical Sciences, University of Geneva, Geneva, Switzerland; Institute of Pharmaceutical Sciences of Western Switzerland, University of Geneva, University of Lausanne, Geneva, Switzerland; Swiss Center of Applied Human Toxicology (SCATH), Basel, Switzerland; Pharmacy Department, Lausanne University Hospital, Lausanne, Switzerland; Center for Research and Innovation in Clinical Pharmaceutical Sciences, Lausanne University Hospital and University of Lausanne, Lausanne, Switzerland; School of Pharmaceutical Sciences, University of Geneva, CMU-Rue Michel Servet 1, 1211 Geneva 4, Switzerland; Institute of Pharmaceutical Sciences of Western Switzerland, University of Geneva, University of Lausanne, Geneva, Switzerland

**Keywords:** container closure integrity, hospital pharmacy compounding, leachable compounds, medical device regulation, off-label use of plastic primary packaging, risk analysis

## Abstract

Hospital pharmacies play a unique role in healthcare by regularly compounding drug products (DPs) in response to hospital demands and practices, for example, drug shortages, to cater to frail and vulnerable patients with infectious, chronic or nutrition-related conditions. Drugs are compounded in precise concentrations for extended durations, sometimes involving complex formulations. A significant challenge in this context is the off-label use of short-term plastic primary packaging for long-term storage of compounded DPs, which could be due to a lack of awareness, financial constraints and inadequate regulation. Without proper risk assessments, such packaging can release potentially harmful leachable compounds, posing a serious threat to patient safety. Evaluating hospital pharmacy compounding procedures to mitigate this risk is essential. While off-label drug use is a well-known concept in hospitals, off-label use of plastic primary packaging is an entirely different practice. In both the United States and Europe, healthcare professionals, including pharmacists, are allowed to use medical devices, including primary packaging, in ways that are not explicitly approved by regulators based on their clinical judgement and best practices, taking into account the patient’s best interest. However, this off-label use could bring about unique risks and challenges, especially in the highly controlled environment of hospital pharmacy compounding, where patient safety is crucial. Therefore, the current review explores the historical context and the current landscape of hospital pharmacies, investigates the potential root causes of container closure integrity issues in pharmaceutical compounding, discusses the materials of construction as well as their physical–chemical properties influencing their roles in most popular primary packaging and finally presents expert opinions aimed at identifying long-term solutions to the existing challenges regarding their off-label uses in hospital pharmacy compounding.

## Plain language summary

A critical opinion-based review of hospital pharmacy compounding with respect to the risk of leachable substances due to the off-label use of plastic primary packaging

1. Aim and Purpose of the Review

The review highlights the risks of off-label use of plastic primary packaging usage in hospital pharmacy batch compounding, that could release potentially harmful leachable compounds. It stresses the importance of testing for these compounds to ensure the safety of frail and vulnerable patients as well as to promote the use of appropriate primary packaging in batch compounding.

2. Context of the Review

Hospital pharmacies sometimes condition long-term storage batches production with primary packaging intended for immediate use. This can be at risk because these devices have not been tested for long-term contact with drug solutions. The main reasons for this practice are a lack of awareness, financial constraints, and inadequate regulations.

3. Organisation of the Review

The review begins with the history and current practices of hospital pharmacy compounding. It focuses on how ready-to-administer compounded drug products and the evolution of container closure integrity-related issues in hospital pharmacy. It also examines why off-label devices are being used, including practical challenges in hospital pharmacies. Additionally, it discusses about materials of construction of common primary packaging used in hospital pharmacy compounding, including the roles of different packaging components. Finally, it offers expert opinions and solutions to mitigate risk, improve safety and propose possible regulatory standards.

4. Proposed Expert Solutions

The review suggests several changes such as improving hospital pharmacy practices to prioritize safety, strengthening regulations and securing more funding for E&L testing, encouraging the wider use of E&L testing in hospital pharmacy batch drug production and developing future solutions to address ongoing challenges in hospital pharmacy compounding.

## Introduction

Medical devices and combined pharmaceutical drug products (DPs) are strictly regulated by various authorities, including the U.S. Food and Drug Administration (FDA) and the European Medicines Agency (EMA); these authorities require thorough testing to ensure specified quality standards within the industry.^1–4^ Pharmaceutical industries follow the Extractables and Leachables (E&L) guidelines and recommendations such as Chapters 1663 and 1664 in the United States Pharmacopoeia (USP) as well as the Product Quality Research Institute recommendations.^5–8^

Ready-to-administer (RTA) compounded DPs are constantly in use in hospitals because of their practical approach for parenteral administration and their cost-effectiveness.^9^ These products are readily available for use in various countries and for adaptation to various administration systems, including syringes, and intravenous (IV) bags. Therefore, owing to their convenience, RTA compounded DPs are being formulated and tailored by hospital pharmacies more often for long-term storage in batches in accordance with the practices and demands of hospital practices and health professionals such as doctors, thus ensuring precise and sterile drug preparation as well as bridging patient–treatment gaps to stay aligned with the current availability on the market.^10–12^

Similar to industries, hospital pharmacies follow good manufacturing practice (GMP) guidelines from the EMA and the FDA to articulate quality expectations for many products as well as to promote different processes.^10–14^ However, medical devices, as in primary packagings, can sometimes be used outside the scope of their regulatory approval. While this practice is legally permissible for health professionals in many countries, it presents significant challenges, including potential risks to patient safety, thus requiring a careful risk–benefit analysis.^1,2^ In hospital pharmacy compounding, one of the most unique concerns regarding off-label use of plastic primary packaging is the release of leachable substances into the DP. Leachable substances are chemicals that migrate from medical devices into compounded medications during preparation, storage or administration. When devices are used off-label, that is, beyond their intended purpose, there is an increased risk of unintended migration and interactions between the compounded drug and the materials used in the devices. Substances, including plasticisers, adhesives or plastic-related degradation products, may have the potential to decrease drug stability, efficacy and safety.^15,16^ In high-risk settings, such as sterile compounding, the presence of leachable substances can be particularly harmful to vulnerable patients.^17^ Therefore, off-label device use must be carefully evaluated in terms of compatibility and potential risks to ensure that patient safety remains a priority while meeting therapeutic needs.^18,19^

The industry already performs E&L risk assessments before market release, which relieves hospital pharmacies from the need to conduct such assessments.^20–24^ Hospital pharmacies cannot manufacture DPs, but they can compound them using primary packaging available in various volumes, as mentioned previously. The compounding approach involves the selection of appropriate primary packaging that are underregulated, even when compounding is performed according to GMP guidelines.^10–14^ However, regarding E&L assessments, hospital pharmacies are not constrained by risk assessments for container closure integrity (CCI)-related matters with respect to the potential migration of leachable compounds and container–content interactions.^17^ Therefore, it would be of best interest to perform a safety risk assessment on hospital-pharmacy-compounded DPs, which are conditioned with off-label primary packaging and involve frail and vulnerable patients, by screening for leachable compounds and performing a toxicological risk analysis in a hospital pharmacy.

This review provides an extensive examination of hospital pharmacy practices, specifically concentrating on compounded DPs and CCI-associated issues caused by the use of off-label plastic primary packaging. The paper is organised into three sections as follows:

1. Historical context: This section examines the evolution of hospital pharmacy practices, tracing the development of RTA compounded DPs and the emergence of CCI-related concerns.
2. Current situation: This section provides an analysis of the present-day situation in hospital pharmacies, examining the potential root causes of off-label use of primary packaging responsible for CCI-related issues in hospital pharmacy compounding.
3. Material of construction (MoC): This section examines the materials commonly used in primary packaging for hospital pharmacy compounding, as well their physical–chemical properties that influence their role as containers. By analysing the composition and properties of these materials, vulnerabilities can be identified that may compromise patient safety, particularly in the context of extended storage.
4. Expert insights and solutions: This section provides valuable perspectives from industry and hospital pharmacy experts to address the existing challenges related to hospital pharmacy CCI-related matters. This section also proposes interesting strategies for enhancing practices and mitigating risks.

## Historical context

### History of RTA compounded DPs and their production in hospital pharmacies

**Figure 1.**
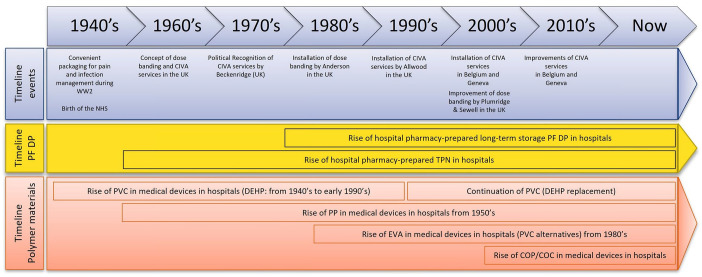
Brief history of the standardisation and centralisation of DPs in hospital pharmacies in Europe. DPs, drug products.

RTA compounded DPs in hospital pharmacies have evolved over the years. Figure 1 shows the history of the standardisation and centralisation of hospital pharmacy DPs in Europe.

DPs have always existed in some form in the context of hospital pharmacies.^9–13,25,26^ The first parenteral nutrition (PN) ever recorded in hospitals occurred in the 1960s.^27,28^ At that time, hospital medical wards had to prepare PN in situ. This approach was fast, but there was an increased risk of standardisation errors. Standardisation is important to ensure that all DPs prepared in medical wards meet the same quality expectations. The concept of standardisation is mostly derived from active pharmaceutical ingredient (API), solvent errors and dosage errors by doctors and nurses. Patients were sometimes administered incorrect DPs at different concentrations, which led to iatrogenic illnesses and complications.^29^ The centralisation of various DPs and dose banding for oncology DPs in hospitals began in the United Kingdom in the 1960s.^30–33^ In parallel, the notion of centralised intravenous additive services (CIVAS) was also developed in the United Kingdom with the aim of centralising the standardised production of RTA compounded DPs in hospital pharmacies and decreasing unwanted contamination and errors in standardisation while promoting rapid treatment.^34^ Dose banding was introduced in the 1980s, followed by CIVAS in the 1990s. These techniques received so much recognition in Europe that Belgium and Switzerland followed the same approach in the early 2000s.^34–37^ Since then, batch DP production in hospital pharmacies has been increasing worldwide. Based on the findings of stability studies that aimed to demonstrate long-term viability, more DPs are being compounded for long-term storage, which would facilitate approaches by hospital practitioners.

### Evolution in plastic primary packaging

The first polymer used in medical containers after the beginning of the 20th century was polyvinyl chloride (PVC), which was designed for blood bags.^38^ Since then, more types of medical containers have popped up. Polypropylene (PP)-based syringes appeared in the 1960s, and ethylene vinyl acetate (EVA) appeared in the 1970s as an alternative to PVC bags.^39,40^ In the early 2000s, cyclic olefin polymers (COPs) and copolymers (COCs) were introduced as innovative high-quality polymer materials by TOPAS^®^, which subsequently commercialised these materials for use as vials and syringes for long-term storage by diverse packaging companies.^9,25,41,42^

### Case studies of CCI-related matters

Numerous cases reported in publications from both industry and hospitals have highlighted issues encountered during drug production and administration. This section presents a series of CCI-related case studies, underscoring the critical importance of testing for E&L-related compounds to ensure patient safety and product quality.

#### PVC-related medical devices

One of the earliest CCI-related cases occurred in the 1970s and involved PVC and different APIs, which caused many different kinds of sorption phenomena. After multiple cases, it was known that lipophilic drugs as well as uncharged molecules are more prone to sorption and have higher interactions with PVC. Di-ethylhexyl phthalate (DEHP), a plasticiser in PVC, was promptly identified as one of the contributors to physical–chemical interactions which can interact with and retain drugs.^43,44^ It was observed to interact with nitroglycerin, diazepam, amiodarone, paclitaxel and many other lipophilic compounds, by adsorbing on the inner walls of the IV bag as well as the IV tubings, thus affecting negatively the efficacy of the DP for the patient.^45–49^ Moreover, there has been cases of proteins, such as insulin, which is crucial in managing the glycaemia of the patient; due to adsorption, it was known to cause loss up to 20%. Finally, lipid-based emulsions are prone to being adsorbed as well affecting the efficacy of the PN for a given patient.^50^ DEHP can also contaminate the drug solution, leading to additional safety concerns. DEHP not only caused physical reactions but also, after patient exposure during treatment, was recognised as causing multiple forms of organ toxicity, such as neurotoxicity, cardiotoxicity, testicular toxicity, ovarian toxicity and hepatotoxicity.^51^ Moreover, the European Chemical Agency (ECHA) scientific committee classified DEHP as an endocrine disruptor in 2006.^52^ Despite all of the information discussed above, some hospital institutions still employ PVC bags and tubing with other DEHP-replacements such as Di-ethylhexyl terephthalate (DEHT), tris (2-ethylhexyl) trimellitate (TOTM) and many others, thanks to its cost-effectiveness compared to other alternatives such as EVA and co-extruded polyolefins.^53–55^

#### Bisphenol A and baby bottles

Bisphenol A (BPA) has been used as a monomer in epoxy resin since the 1950s, and a decade later, the first baby bottles were made of BPA-based polycarbonate (PC) material.^56,57^ Over time, it has been shown that BPA is an endocrine disruptor, bearing similarity to the molecular structure of oestrogen. The endocrine-disrupting nature of BPA gained political recognition from the ECHA in 2017.^58^ After being infamous, BPA was replaced by other bisphenol derivatives, such as bisphenol F and S; however, these compounds were recently discovered to have endocrine-disrupting potential, raising similar health concerns as BPA and are more resistant to degradation, leading to potential environmental persistence.^59–61^

#### Silicone oil interactions

In the 1980s, several incidents occurred involving the contamination of insulin solutions in syringes with silicone oil. This effect could have been driven by the potential interaction of different amino acid groups with lipophilic side chains on the protein with silicone oil.^62,63^ This could also be an issue since insulin is a drug with a narrow therapeutic index. Moreover, there were numerous cases of silicone oil droplets (as plunger head lubricants) intravitreally injected into patients’ eyes as floating bodies due to possible off-label use of medical syringes.^64,65^ While off-label use of primary packaging is not uncommon in hospitals, this was one of the first published reports on this topic.^66^ Moreover, since the 1980s, it was known for insulin to interact, via adsorption with polyolefins, such as polyethylene (PE), in hospital medical wards, affecting the efficacy of the drug. After numerous reviews, a change in medical packaging to COC was deemed necessary step to avoid CCI-related matters involving insulin.^67,68^

#### Tungsten and acrylic acid interactions

Tungsten, commonly used in manufacturing the needles and barrels of prefilled syringes (PFS), has been associated with interactions with biologic drugs. Tungsten is used during the syringe manufacturing process to create the fine bore of needles and PFS barrels. Tungsten pins are inserted into glass syringes during the moulding process to maintain the bore’s shape. Therefore, trace amounts of tungsten can leach into the DP from these components, leading to interactions with biologics. As a consequence, these trace amounts can cause protein aggregation, which could lead to compromised drug efficacy and increased immunogenicity risks. Other than tungsten, acrylic acid derivatives, also known as the glue component responsible for attaching the needle to polymer piece as well as on the glass syringe barrel, can leach and interact with the biologics via protein aggregation.^69–71^

#### Eprex^®^ and red blood cell aplasia

Furthermore, there are a variety of CCI-related cases involving proteins and diverse forms of pharmaceutical packaging. One example is the case of Eprex from Janssen-Cilag^®^ (Epoetin-Alfa), which caused an immune response in many patients in the late 1990s, leading to decreases in haematocrit levels (i.e. percentage of red blood cells in the blood). This immune response was due to a change in the protein stabiliser from albumin to polysorbate 80, which caused rubber-related compounds from the plunger head of the syringe to leach into the solution.^72,73^

#### Rubber-related mercaptobenzothiazole

Speaking about rubber like vial stoppers and disposable plunger heads, a carcinogenic leachable compound was identified as 2-mercaptobenzothiazole (MBT), a vulcanisation agent as early as the 1980s. These related compounds were known to cause immunogenic response in patients. As a consequence, rubber manufacturers have switched to low-extractable elastomers for stoppers and plunger heads and implemented stricter compatibility studies.^74,75^

#### Stainless steel interaction with biologics

In terms of manufacturing, in early 2010, leachable compound interactions were made aware during production. Stainless steel surface of the production equipment can interact with the biologics in a way that metal traces of iron, chromium and nickel, being major components of 316 L stainless steel, were leached into solution, influenced by the formulation. Moreover, stainless steel can cause of aggregation of a monoclonal antibody, induced by its adsorption.^76,77^

Throughout the years, hospital pharmacies have experienced substantial cases of CCI-related issues. Industrial and hospital pharmacies have so much in common in terms of CCI-related issues because most pharmaceuticals manufactured for the market are used on patients by hospital practitioners/caregivers. After observing these series of CCI-related events, these historical cases underscore the critical importance of CCI considerations in pharmaceutical packaging. Hospital pharmacies are strongly urged to acknowledge and address these potential concerns, especially if batch-compounded DPs are involved, to ensure patient safety and maintain product integrity.

## Current situation in hospital pharmacies

### Reasons to compound RTA DPs in hospital pharmacies

There has been an increase in the use of RTA DPs, including a diverse range of active therapeutic products (ATPs) used for acute and chronic treatments at different concentrations for different applications, such as anti-infectious DPs, cardiovascular DPs, anaesthetics, muscle relaxants, insulin, PN, chemotherapy and antidotes.^34,37^ Hospital practices vary from hospital to hospital and benefit from the market availability of RTA compounded DPs. Some RTA DPs may need to be compounded when market-based RTA DPs are unavailable owing to temporary shortages or permanent market withdrawal. Hospital practitioners could switch from one API to another, but, sometimes, there is good evidence that the ATP switch is not a viable option. Therefore, hospital pharmacies tend to recreate withdrawn DPs to bridge gaps in potentially uninterruptable treatments.^78,79^

Hospital pharmacies could also compound DPs according to specific hospital practices, which consist of implementing approaches involving the same or different API(s), at a particular concentration, with simple or complex excipients, for a particular set of patients. The compounding of DPs could be helpful for certain specialised hospital departments for specific patients, who may require more attentive medical care. These different medical wards mostly involve adults but may also involve preterm infants, neonates and paediatric patients. Some treatments are selected based on patient weight, thus challenging practitioners who are searching for commercially available RTA DPs based on market standards. Therefore, hospital pharmacies are required to compound these DPs in response to demands by considering the specific API(s) and their concentration(s).^34,80,81^

The possibility of compounding DPs for enough patients who may want to leave the hospital but may also resume home-based parenteral chronic treatment may be a further argument for hospital compounds, for example, anti-infectious preparations, where the DP is stable outside the hospital.^82,83^

### Safety concerns in hospital pharmacy compounding

First, according to legal aspects, industries are required to perform a battery of tests on medical devices and pharmaceutical DPs, such as E&L experiments and toxicology.^1–7^ For hospital pharmacies, in contrast, the legal requirements for assessing the safety risk of primary packaging containing compounded DPs are minimal, given that none of the products are intended for the open market and that all compounding batches possess a limited expiration date of 1 year, depending on the stability of the DP, regulated by authorities in Europe and the United States.^12,13^ Because CCI-related cases have occurred since the first use of medical batches in hospitals, it seems logically pertinent to promote E&L testing in hospital pharmacies alongside CCI monitoring.^17^ Therefore, evaluating plastic-related compounds in hospital-pharmacy-prepared DPs would be beneficial.

### Off-label use of plastic primary packaging

A suitable definition would be the use of plastic primary packaging intended for one purpose but repurposed for another. For example, plastic containers designed for drug administration for long-term storage have been used. This poses a problem because devices intended solely for administration have not undergone the necessary risk assessments, such as industrial E&L evaluations for long-term storage. This could be considered off-label use. Although off-label use of medical devices, including primary plastic packaging, may be permitted by health professionals in hospitals such as pharmacists, these problems can occur frequently when careful risk–benefit analyses are not performed in hospital pharmacies owing to a lack of awareness, financial constraints and/or inadequate regulations.

#### Lack of awareness

This knowledge gap can result in a significant mismatch between what suppliers offer and what hospitals actually need. There are notable differences in scale and practice when it comes to hospital pharmacies. In terms of scale, the production and consumption of centralised treatments in hospitals is relatively small by industry standards but significant within hospital operations. Furthermore, there is a sharp contrast between the large number of medical devices used for bedside preparation and those used for centralised production in hospital pharmacies. As a result, suppliers often focus on providing medical devices designed for use in ward units, overlooking the specific needs of pharmacy production. Suppliers typically have a limited understanding of hospital pharmacy practices and tend to focus on devices designed for immediate use (24, 48 or 72 h). However, the challenge in pharmacy practice is the long-term storage of treatments, which may extend up to a year or more. For such prolonged storage, primary packaging needs different characteristics than those used in short-term applications. Hospital pharmacists have not extensively tested primary packagings for long-term storage regarding E&L-related issues.

Additionally, hospital pharmacists face challenges in staying informed about current primary packaging suitable for long-term storage, as these packaging solutions are designed primarily for industrial filling and storage of drug solutions rather than for hospital pharmacy-compounded drug production. Therefore, there is indeed a lack of suitable hospital pharmacy-compatible primary packaging on the market. Consequently, there is a mutual misunderstanding between the industry and hospital pharmacies regarding the appropriate containers for storage and administration, which could ultimately compromise patients’ safety. As long as this issue persists, instances of unintentional off-label of containers in hospital pharmacies will continue to occur.

#### Financial constraints

According to hospital pharmacies, there are several advantages to using immediate-purposed packaging for long-term storage, such as its readily available sterile packaging in individual units and the convenience of purchase, which makes them easily handled in clean rooms. Therefore, these products are the least costly for hospital pharmacy-level production. Hospital pharmacies constantly rely on manual workforces to perform semi-technical and pharmaceutical labour. If a strategy involving long-term packaging was implemented, investments in suitable filling and packaging equipment for batch production would be practical. However, not all hospital pharmacies can possess up-to-date and expensive automated production equipment. Efforts to procure these production tools may increase costs, which may include maintenance and qualifications for further hospital pharmacy DP batch production. Importantly, specific small-batch product could be relatively sophisticated and difficult to compound. In hospital pharmacies, a catalogue containing a large variety of DPs would be useful for many medical departments; however, the hospital pharmacy production unit would need to be versatile, adaptable and agile. Furthermore, if ad hoc equipments were installed in clean rooms, rigorous and time-consuming procedures would need to be established to ensure compliance with GMP guidelines. Therefore, despite the inappropriate use of administration-purposed packaging for long-term use, hospital pharmacies frequently employ these solutions for financial and practical reasons. Long-term packaging is convenient from a security point of view, but it may come at a higher price in terms of purchasing materials, filling equipments and performing maintenances.

Moreover, most long-term primary packaging providers normally sell them to the industry in bulk due to the high scale of manufacturing. Bulk purchases could be an inconvenient source of waste in hospital pharmacy DP production because of their smaller-scale production.

Historically, primary packaging has been made of easily reusable and sterilisable glass materials. However, glass containers are difficult to maintain because they are heavy to transport, less practical during administration and not impact resistant. Therefore, plastic containers made of PVC, PP, PE, EVA, etc., which are lightweight, cost friendly and shatterproof, have become popular and viable options in hospitals worldwide. Plastic containers were used for both DP administration and long-term storage. As time passed, better plastic containers suitable for long-term storage, such as COP and COC materials, became available on the market and are currently employed by the industry for market release. However, in hospital pharmacies, these old practices have remained in place because new containers for hospital-pharmacy-compounded DPs would require new stability studies, which would be costly and time-consuming.

#### Inadequate regulation

To date, no clear or unified regulatory framework exists that addresses the use of off-label primary packaging in hospital pharmacy compounding worldwide. European Medical Device Regulation (Regulation (EU) 2017/745, i.e. MDR) and FDA regulations focus primarily on the intended use of medical devices labelled by manufacturers.^1,2^ These regulations ensure that medical devices meet specific safety and effectiveness criteria when used as approved, but they do not provide comprehensive oversight for their use beyond these approved indications. For pharmaceutical combined DPs, industries are required to perform risk assessment via E&L analysis of new drugs for market entry.^5–8,20–24^ Compared with pharmaceutical DPs, medical devices are often approved for specific purposes under particular conditions.^1,2^

A serious concern regarding off-label use of primary packaging in hospital pharmacy compounding is the lack of required testing for E&L-related compounds that can migrate from primary packaging materials into the compounded medication. According to the European and FDA MDR, health professionals, including doctors and pharmacists, are allowed to employ off-label medical devices, to be used as a combined container closure system or a DP based on their clinical judgement and best practices.^1,2^ However, when these devices are used off-label, no suitable material testing is currently required to confirm contact compatibility with specific drug formulations, therefore stepping outside the boundaries of regulated and approved uses. This lack of regulation and oversight brings about possible significant risks, particularly when frail and vulnerable patient populations are involved, like neonates, geriatric patients, or those with compromised immune systems, being administered frequently over a long-term basis. These devices may not be chemically compatible with the compounded drugs they are storing or delivering. This can lead to drug degradation caused by interactions with incompatible materials. There is a lack of comprehensive data on how materials respond to different solvents, pH levels or temperatures when used outside of their intended function. Therefore, hospitals and healthcare professionals are left responsible for ensuring the safety of off-label medical device applications. Without regulatory mandates for compatibility testing in off-label uses, there is no guarantee whether these devices would maintain the compounded medication’s efficacy or safety over time.

Compounded DPs are not tested via E&L analysis, unlike industrially manufactured pharmaceutical DPs. Therefore, potentially harmful leachable compounds may contaminate compound DPs over time, thus affecting patients. This risk exists because hospital pharmacy compounding often involves small batches of highly customised medications, which are not subjected to the same rigorous regulatory scrutiny as mass-manufactured pharmaceuticals. Therefore, it remains unclear whether compounded DPs are truly less risky. Patients receiving compounded medications are a subset of the general population, and their safety should be restricted to equally stringent standards.

Moreover, various publications and legal documents from Europe highlight that incorporating container–content interaction-related testing into stability studies is regarded as a best practice to ensure the efficacy of hospital pharmacy batch-compounded DPs in hospital pharmacies. While container–content interaction testing can provide critical insights into the presence of leachable compounds that may interact with DP components, for example, APIs and excipients, it is important to recognise its limitations. Not all leachable compounds will interact with the DP, and some may pose potential harm independently. Therefore, it is essential to go beyond container–content interaction testing and conduct comprehensive E&L studies to perform risk assessments. These evaluations offer a broader understanding of potential risks, providing strong evidence to guarantee the safety of compounded DPs for hospital patients.^34,36,84,85^

The absence of regulation also poses ethical and legal concerns for healthcare professionals. While these devices may be necessary to bridge a patient treatment gap, especially for frail and vulnerable patients such as neonates, infants, geriatric and immunocompromised patients, it also imposes an ethical responsibility upon the hospital and its healthcare professionals to ensure safety in absence of regulatory framework, whether through the off-label use of primary packaging or its appropriate application in conditioning batch-compounded DPs for hospital patients. The absence of a comprehensive regulatory framework also creates a legal grey area. It remains unclear to this day whether the hospital, the healthcare provider or the manufacturer of the device should be held accountable for adverse events or complications due to the use of off-label medical devices. Moreover, the absence of required reporting obligations for post-market surveillance for off-label use of device could complicate efforts to track adverse events, thus being a potential obstacle in data gathering that could be crucial for future regulatory changes. Furthermore, there could also be a potential disconnect between regulatory bodies and the realities of hospital pharmacy practice. Regulatory agencies may not fully grasp how off-label devices are being used in these settings and the unique risks posed to vulnerable populations. This lack of understanding could contribute to insufficient oversight and unclear guidelines on the use of off-label devices in compounding.

### Inappropriate use of labels, scale graduations and secondary packaging

Another form of improper use of medical devices is labelling. It is, of course, the right approach for both industries and hospital pharmacies to label a DP to avoid confusion and to direct the user and the nursing staff/medical practitioners in proper administration. In industry, when a primary packaging is made of a polymer material, it is crucial to adopt a low-extractable label, which was studied/evaluated beforehand via E&L studies, to test its compatibility with primary packaging. However, in hospital pharmacies, untested labels are selected for use on primary packaging, which could promote the migration of label-related compounds into the drug solution.

In addition to labelling, graduation markers on plastic primary packaging could also be an issue. In industry, primary packaging for long-term storage is not imprinted with graduation markers but is instead labelled with low-extractable labels. The use of primary packaging solely for administration involves graduation markers that have not been assessed on a long-term basis. During long-term storage, both labels and graduation marks could promote the migration of potentially harmful substances such as acrylate-related adhesives through the material and into drug solutions.^15,16^

**Figure 2.**
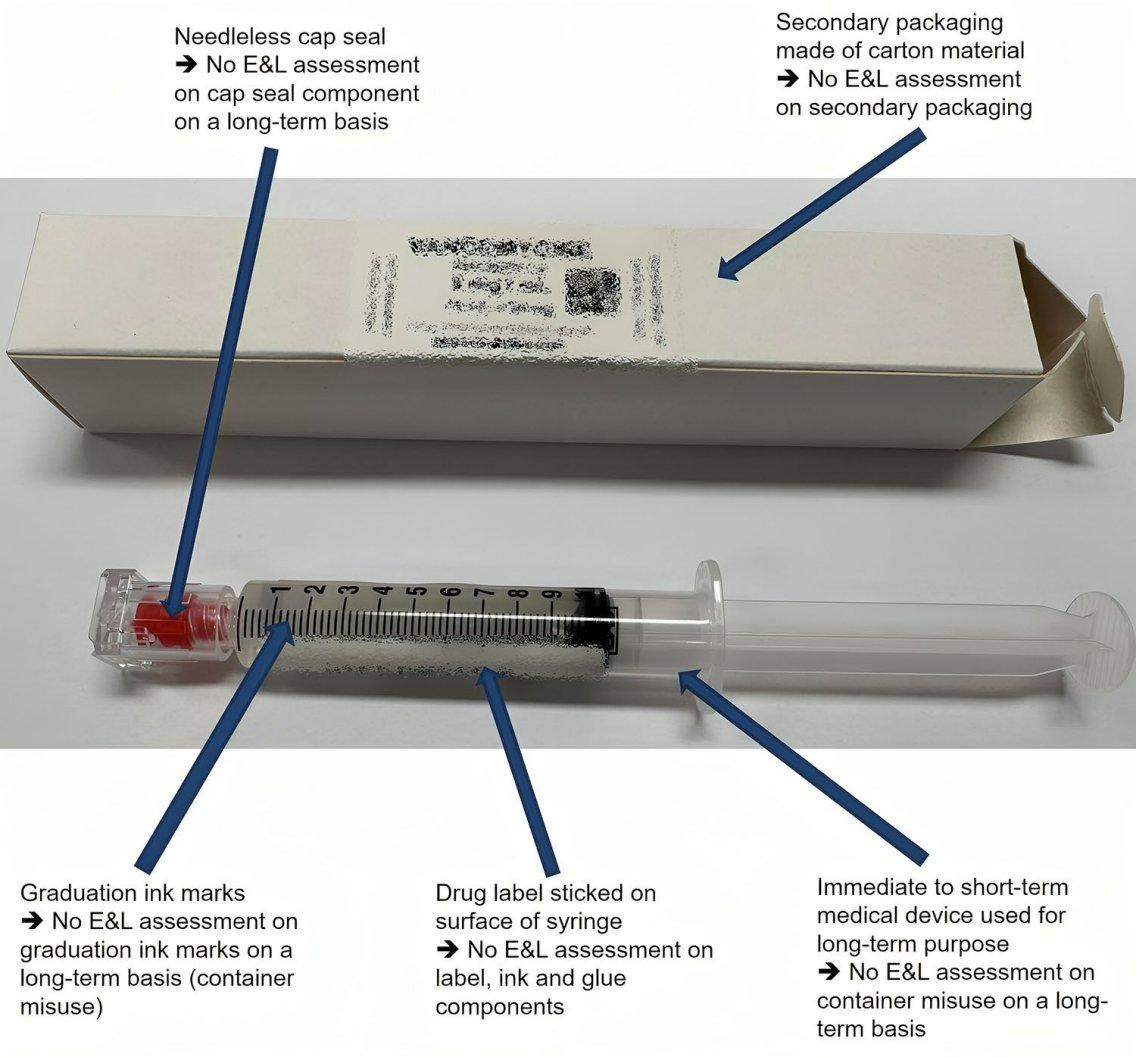
Example of long-term stored hospital-pharmacy-compounded DPs with descriptions of different points of medical device misuse.^15,16^ DPs, drug products.

Finally, selecting appropriate secondary packaging is crucial. In hospital pharmacies, secondary packaging is not evaluated beforehand, unlike in industries. Sometimes, cardboard material is used for packaging, which could pose a problem since it could promote the release of paper residues such as abietic acid derivatives into the drug solution.^5,86^ Therefore, proper selection of secondary packaging is also important. Figure 2 shows the different points of medical device misuse on a real hospital-pharmacy-compounded DP.

### Other factors increasing the risk of CCI-related issues

DPs mainly concern high-risk patients, such as preterm infants, neonates and children. These patients do not possess a developed immune system, often cannot efficiently eliminate toxic compounds and are thus more vulnerable to potential endocrine disruptors, for example. Compared with adults, neonates and infants have important anatomical and physiological differences.^87^ Their bodies are undergoing development, which makes them more vulnerable to toxicological hazards when exposed to some categories of chemicals.^88^ Hospital pharmacy-prepared products for moderate-to-severe chronic infections and metabolic illnesses and PN are high-risk products for these underdeveloped patients, especially when frequent administration, specific APIs, specific concentrations and compatible formulations are needed. In the current market, RTA DPs for these patients seldom exist due to low market demand.

Moreover, the application of parenteral and ophthalmic administrations requires more attention in the preparation of DPs since the risk is greater due to the need for strict sterility and stability requirements. Contaminants or improper formulations can lead to severe adverse effects, such as infections or irritation, thus making these routes of administration particularly sensitive. The off-label use of syringes, especially for applications beyond their intended purpose, can pose a significant risk to sterility and effectiveness. One potential concern is plunger head shrinkage, which can compromise the seal between the plunger and syringe barrel. Loss of seal integrity may result in microbial contamination and fluid leakage, jeopardising both the safety and integrity of the DP. Additionally, functional alterations such as uneven or erratic plunger movement can cause difficulties in drug withdrawal or injection, increasing the potential for dosing errors. These risks may be exacerbated by sterilisation methods such as autoclaving, though such practices are generally not recommended for disposable syringes. While no published reports have explicitly documented these issues, they remain plausible scenarios, as discussed by industry E&L experts.

Formulations are crucial factors that may increase the incidence of CCI-related issues. DP formulations such as suspensions, all types of emulsions, micellar solutions and protein-based solutions are prone to plastic-related compound extraction from medical containers because of the presence of surfactants, protein stabilisers and diverse functional groups on the protein.^89,90^ This risk is concerning for any patient who has to undergo chronic treatment.

### Examples of hospital pharmacy compounding and off-label primary packaging

Several examples have been compiled to illustrate different batch production compounding processes that involve off-label primary packaging. Typically, most compounding involves simple matrices, such as water for injection, normal saline or low-concentration buffer solutions, often with pH levels ranging from acidic to neutral. However, some compounding processes use more complex matrices, which may present higher risks. Below are examples of compounded DPs that could potentially lead to the migration of leachable compounds into the drug solution, a concern arising from the use of off-label primary packaging.^15,16^

#### RTA PP syringes containing vancomycin 5 mg/mL

For RTA PP syringes, the active DP is antibiotics for neonates and children. One example would be the use of vancomycin over a period of weeks and months to treat invasive staphylococcal infections (MRSA: methicillin-resistant *Staphylococcus aureus*).^90–92^ The syringe utilised in this case is a BD^®^ Plastipak^®^, employed in the conditioning of vancomycin, with a long-term stability of 6 months. This usage could be classified as an off-label application of primary packaging. These syringes are primarily designed for immediate or short-term use in syringe pumps to administer saline solutions or DPs. Notably, it could raise potential concerns about its compliance with safety and quality standards.^15,16^

#### RTA EVA IV bag containing PN

EVA IV bags are normally used for PN for neonates and children. Lipid components are included in piggyback administration (administered separately). The solution normally contains a series of salts, glucose and amino acids. It is administered to appropriate paediatric patients whose gastrointestinal tract is unable to support normal growth and development.^70^ PN can be given to infant patients before or after surgery to bypass the mouth in cases of acute or chronic gastrointestinal malfunction to increase caloric intake.^93–95^ The IV bag, made of a single-layer EVA polymer, is used for conditioning PN. Single-layer EVA is typically intended for short-term use, which makes this application an off-label use of primary packaging. Additionally, the PN was evaluated for long-term stability over a 12-month period. This raises potential concerns regarding the compliance of the packaging with established safety and quality standards.^16^

#### RTA cyclic olefin copolymer vials containing insulin 1 IU/mL

RTA COC vials were supplemented with diluted insulin (NovoRapid^®^, NovoNordisk) at 1 IU/mL, which was adapted for neonatal administration in cases of postnatal hyperglycaemia and hyperkalaemia.^96,97^ The vial in question is constructed from COC material, which is specifically designed for extended contact with drug solutions and offers low susceptibility to CCI-related issues. However, it was noted that the label on the vial had not undergone E&L assessment, leaving its low extractable profile unverified. While the stability of the DP was determined to be 12 months based on studies, the prolonged contact of the label with the vial surface raises concerns. Over time, there is potential for migration of compounds through the vial material into the drug solution, posing questions about the packaging’s compliance with established safety and quality standards.^16^

#### RTA PP syringes containing insulin 1 IU/mL

PP syringes were used to condition insulin at 1 IU/mL, similar to the previous case, which could be adapted for neonatal administration in cases of postnatal hyperglycaemia and hyperkalaemia. A stability evaluation of this injectable was conducted, compounded from two branded products, Huminsulin^®^ Normal 100 and Actrapid^®^ Penfill^®^, diluted with 0.9% NaCl solution and conditioned in 50 mL disposable plastic syringes (Original-Perfusor^®^ syringe or BD Perfusion syringe).^96–98^ Notably, these syringes are intended for immediate use, such as administration or withdrawal, and not for prolonged contact with drug solutions like long-term storage. It is known for insulin to interact with PP syringes through adsorption, which can potentially affect the stability of the drug solution over time.^68,99^ To counter this interaction, COC- or COP-based syringes would be most recommended for batch production intended for long-term storage. These advanced polymer materials offer superior compatibility with the drug solution, significantly reducing the risks of adsorption.^16^ Additionally, transitioning to COC or COP syringes may enable stability studies to demonstrate longer storage durations due to the enhanced container–content compatibility. This example of a case study proves the importance of ensuring compliance of the packaging with rigorous safety and quality standards to safeguard the efficacy and integrity of the DP.

#### RTA PP syringes containing ketamine HCl

An international alert from BD has highlighted the potential for interactions between certain molecules and some of their syringes. The case study involved the use of 3 mL BD syringes, to store ketamine HCl, as a means of CIVAS. The concerned institution aimed to evaluate any possible interaction between ketamine and these syringes. Findings have shown that ketamine in disposable syringes remained stable for at least 50 days without CCI-related issues with the syringes.^100^ However, it is important to note again that there is a case of off-label use of primary packaging, as they are intended for ‘general purpose injection and aspiration of fluids from vials, ampoules and parts of the body below the surface of the skin’ according to BD Plastipak technical sheets. While no interaction was observed in this study, it does not eliminate the possibility of migration of potentially harmful leachable compounds from the syringe components and a toxicological risk assessment should be highly recommended for the sake of patients.^4,15,16,101^ These compounds have not been evaluated under long-term storage conditions, which raises concerns about the safety of using off-label primary packaging, especially in the absence of comprehensive E&L assessments.

#### RTA EVA IV bag containing PN

Standard PN solutions are complex mixtures of interacting components that can degrade over time. The physicochemical and microbiological stability of a hospital pharmacy-prepared PN formulation specifically designed for neonatology was investigated. Results have shown that the PN remain stable for up to 4 months when stored at 2°C –8°C, ensuring their safe administration to preterm infants.^102^ Regarding the primary packaging, the PN was conditioned in an EVA IV bag from Baxter. However, it remains unclear whether the bag is composed of monolayer or multilayer material. If the bag is monolayer, this could constitute an off-label use of the packaging, intended for short-term use (weeks) rather than extended periods (months). On the other hand, if the bag is multilayer, it would likely be suitable for long-term storage. Given that this formulation involves preterm infants and neonates, both highly frail and vulnerable populations, an E&L assessment should be highly recommended. Performing such an evaluation would be essential to assess the safety of the product for patient use, ensuring that no harmful leachable compounds migrate from the packaging material into the drug formulation.

#### RTA PP syringes containing bevacizumab

This study was established to compare and assess the storage stability of compounded bevacizumab in PC and PP syringes over a 6-month period. Bevacizumab is a monoclonal antibody, approved for IV infusion to treat diverse cancers like metastatic colorectal cancer, non-small cell lung cancer, renal cell carcinoma and glioblastoma. In this case, this API was not licensed for intravitreal injection to treat retinal diseases.^103–105^ As a result, no significant difference in the quality of bevacizumab repackaged into prefilled PC and PP syringes was observed when compared with bevacizumab supplied directly from the vial over the 6-month storage period. It is likely that these syringes used were BBraun^®^ Omnifix^®^ and BD^®^ Plastipak^®^, as these are commonly marketed by these companies. Again, these syringes are labelled for immediate use, and no E&L assessments were conducted as part of risk assessment for long term storage to ensure patient safety. Therefore, while the stability of the bevacizumab product was not compromised during the 6-month storage, the lack of comprehensive testing for leachable compounds raises concerns about the long-term safety of this compounded formulation.

Although more relatable publications exist, many are difficult to evaluate due to missing critical information, particularly the commercial names of polymer materials used in primary packaging and the lack of transparency regarding their inventory. This lack of detail significantly complicates the assessment of potential risks associated with batch compounding practices in hospital pharmacies worldwide. Furthermore, reports on leachable assessments for live hospital pharmacy compounding batch production are rarely available through publications or inquiries.^15–17,106^ This is largely because current guidelines do not mandate E&L assessments as part of patient safety quality testing.

As highlighted, the off-label use of primary packaging, coupled with the absence of E&L assessments for batch compounding, can indirectly jeopardise patient safety, particularly under current compounding practices. Without comprehensive regulations to mitigate these risks, the well-being of frail and vulnerable patients, who frequently require repeated administrations and long-term treatments, remains compromised.

## MoC in hospital pharmacy batch primary packaging

This section examines the MoC used in primary packaging, such as syringes and IV bags, which are essential in hospital pharmacy batch compounding operations. Alternative materials commonly used in the pharmaceutical industry are also highlighted, emphasising their superior performance, enhanced safety and regulatory compliance.^107^ Additionally, the role of each packaging component is analysed, focusing on how material properties influence functionality, durability and the overall safety of DPs.

### RTA syringes

RTA syringes are increasingly batch compounded in hospital pharmacy due to their numerous advantages, including enhanced safety, efficiency and overall healthcare quality. These syringes are used for a wide range of DPs such as anti-infectives, chemotherapeutics, anaesthetics and analgesics, emergency and critical care medications, sedatives and many more.^108^

However, as previously noted, disposable PP syringes, for immediate use, are often employed off-label for batch production based solely on stability assays, without conducting E&L testing.^15,16^ These syringes are popular for several reasons: they are convenient, cost-effective, pre-packaged individually and pre-sterilised. Examples include BD^®^ Plastipak^®^ and BBraun^®^ Omnifix^®^. While made of medical-grade polymers, these syringes are primarily designed for immediate or short-term use via syringe pumps and to withdraw and to administer saline solutions and DPs for bedside treatments. Their labelling reflects toxicological assessments and technical specifications, not suitability for long-term storage.^1–4,109–112^

The introduction of COC and COP syringes has changed the way the industry operates. Pharmaceutical companies now rely on syringes like BD Sterifill^®^ and Schott^®^ TopPac^®^, especially designed for long-term storage, to condition batch DPs, and these syringes should be considered a recommended option for conditioning industrial pharmaceutical injectables as well as hospital pharmacy batch productions. These specialised syringes are clearly labelled, by manufacturers, as ideal primary packaging for long-term contact with drug solutions because of their superior quality, low extractable profile and compatibility with a wide range of DPs.

Prior to the introduction of COC and COP syringes in the market, glass-based syringes made of glass material were and still are widely used by pharmaceutical companies for packaging injectables. Being of USP/EP Type I borosilicate glass quality, they are compatible to a large array of compounds. However, glass also has its downsides, such as fragility and weight, which can limit its practicality in some situations. These limitations have driven industries and hospital pharmacies to turn to COC and COP syringes as ideal primary packaging alternatives.^9,112–118^ In the following sections, different components of the syringe will be discussed in terms of the materials used and their specific roles.

#### Barrel

Barrels of disposable syringes like BD^®^ Plastipak^®^ and BBraun^®^ Omnifix^®^ are made from PP, a semi-crystalline thermoplastic isotactic polymer derived from propylene monomers. Thanks to its semi-crystalline morphology, the barrel possess interesting physical properties, including a right balance of flexibility and hardness, as well as being lightweight, ensuring ease of utilisation of the syringe during withdrawal and administration of a liquid. Apart from that, it is proven to be compatible to a wide range of drugs, including aqueous and lipid-based solutions. The semi-transparent nature of this material allows the user to perform visual inspection of its contents, while its hydrophobic properties provide excellent dimensional stability in humid environments. Furthermore, the barrel’s smooth surface ensures high compatibility with medical-grade silicone oil, enabling frictionless plunger movement.^118–122^ Despite all advantages, the material of the barrel is exclusively limited for immediate use. Any deviation from their intended purpose should be reconsidered for a change in a primary packaging with a more appropriate material.

In contrast, syringe barrels coming from BD^®^ Sterifill^®^ and Schott^®^ TopPac^®^, manufactured using COP and COC, respectively, are specifically designed as highly performant primary packaging, purposed for long-term storage, particularly for RTA DPs. These high-performance amorphous thermoplastics, derived from norbornene monomers, demonstrating unique advantages for both pharmaceutical and medical applications. Barrels, made from these materials, offer excellent physical and chemical barrier properties, including good moisture resistance, excellent gas impermeability, UV protection, high physical impact resistance, dimensional stability and compatibility with multiple sterilisation methods. Additionally, they prove to possess optimal compatibility with diverse drug formulations, including small molecular drug compounds, biologics and vaccines. They have been tested to have a low extractable profile, minimising risks of contamination from leachable compounds due to long-term drug-material contact, therefore ensuring patients’ safety. They are optically transparent, allowing operators to clearly inspect for particulates and discoloration, unlike the previous syringes. Upon reception, COP and COC syringes are typically delivered in dismantled, sterilised packaging, facilitating compatibility with automated filling and assembly equipments.^9,112–116,118–123^

For glass PFS, the barrels are made from USP/EP Type I borosilicate glass, recognised for its superior chemical inertness. This medical-grade glass material is non-reactive; therefore, it is ideal for maintaining drug purity and stability. It offers excellent mechanical and thermal stability, ensuring suitability for a variety of pharmaceutical processes. Thanks to these interesting properties, it is approved in the pharmaceutical industry, meeting stringent regulatory standards. However, as a major downside, the fragility and weight have led many pharmaceutical manufacturers and hospital pharmacies to transition toward COP or COC syringes, although these polymers remain to be higher cost than glass material. These alternatives provide a much safer, more durable and cost-effective approach for modern pharmaceutical DPs.^9,116–118,123^

#### Scale graduation

The scale graduation on barrels of disposable PP syringes, like BD^®^ Plastipak^®^ and BBraun^®^ Omnifix^®^, is printed using black and white UV-curable inks, which solidify upon UV exposure. Once cured, it adheres well on the surface of the barrel and resists to diverse physical and chemical phenomena, including wear and rubbing during use, as well as exposure to different medical solvents, and drug solutions, making it highly suitable for hospital setting. Furthermore, the scale graduation enables healthcare providers to measure, withdraw and administer liquid from vials and bottles for bedside treatments. In terms of toxicity, the ink is tested to possess a low extractable profile, ensuring low risk of contaminations, thereby assuring patients’ safety. However, this low profile is maintained for as long as the syringes are used as intended: for immediate use and not exposed to extended storage.

Syringes like BD^®^ Sterifill^®^, Schott^®^ TopPac^®^, and glass syringes do not feature pre-printed scale graduations on their barrels because they are designed to deliver a controlled, full-dose injection volume and are not intended for partial administration. However, if a partial volume administration is required, then scale graduations in the form of a label can be added externally, which can be printed, adhered or shrink-wrapped to the barrel surface. Labels can be customised for specific drugs at different concentrations to aid health providers in their treatments as well as out of hospital patients. These labels must be ascertained to possess a low extractable profile, to minimise contamination of DPs, ensuring as a result patient safety. This issue does not apply to glass syringes as label-related leachable compounds cannot migrate through the material into the DP.^9,109–111,116,124–127^

#### Plunger rod

Plunger rods, from disposable PP syringes like BD Plastipak and BBraun Omnifix, are also made of isotactic PP and are not in contact with the drug solution. It is a critical component engineered for smooth operations during medical applications. It must be lightweight and durable to ensure strength and rigidity for the push action of the syringe. The smooth movement of the rod must maintain seal integrity, thus preventing leaks during operation. The rod motion enables volume measurement and withdrawal of fluids, which are critical in applications like medication administration, blood collection and laboratory use.

The plunger rods, from BD^®^ Sterifill^®^ and Schott^®^ TopPac^®^ and glass syringes, are also made of PP and are built to possess the same purpose as the plunger rods from PP syringes like BD^®^ Plastipak^®^ and BBraun^®^ Omnifix^®^. The primary differences are that these syringes are designed for more precise and delicate movements and are supplied already dismantled from the main syringe body and the plunger head. During the filling process, all components are assembled through the filling equipment to create a complete PFS. Furthermore, separating the plunger from the main syringe body is also advantageous for assembly into secondary packaging, as most pharmaceutical companies adopt this approach to ensure the optimal use of space.^9,109–123^

#### Plunger head

The plunger heads from disposable PP syringes like BD^®^ Plastipak^®^ and BBraun^®^ Omnifix^®^ syringes are built using thermoplastic elastomers, like silicone or butyl rubber. These materials are latex-free, making it ideal for latex-related allergies. The plunger heads are tested non-toxic, non-pyrogenic and biocompatible. They serve two main purposes: to form an airtight seal with the syringe barrel, ensuring sterility and preventing leakage, as well as to facilitate the withdrawal and administration of liquid into and out of the barrel respectively, thanks to the silicone oil slip agent. Moreover, the material is physically durable and chemically resistant, for short-term compatibility with a wide range of solvents and drug solutions.

In contrast, syringes, such as BD^®^ Sterifill^®^, Schott^®^ TopPac^®^ and glass syringes, feature plunger heads as separate components that screw onto the plunger rod to form a unified structure. These plunger heads are made from higher quality butyl isoprene rubber, which are purposed for long-term contact with DPs. This is due to its excellent chemical resistance and low permeability, ensuring superior moisture and gas barrier properties to maintain drug stability. The plunger head rubber material possesses a low extractables profile, assuring low contamination risks and patient safety. The silicone oil slip agent used with these plunger heads is specially formulated to require smaller amounts while still ensuring optimal performance, unlike the plunger heads used in disposable PP syringes. Furthermore, they can withstand different sterilisation processes such as autoclaving, ethylene oxide and gamma irradiation. For enhanced functionality, the rubber material can be teflonised, incorporating a fluoropolymer layer that provides additional protection against harsher matrices such as lipid emulsions and proteinic solutions. This advanced feature enhances the plunger’s suitability for specialised drug formulations, further supporting safety and efficacy in medical applications.^9,112,128–131^

### IV bags

Hospital pharmacies commonly rely on IV bags for compounding PNs and other DPs. These bags are typically constructed from materials such as PVC, EVA and co-extruded polyolefins, all of which are approved by manufacturers for nominal, short-term preparations requiring a doctor’s prescription. These applications usually last between 24 and 72 h. However, in some hospital settings, these IV bags can be repurposed for batch production, particularly for general PN formulations and DPs intended for specific patient groups, such as neonates or paediatric patients. In such cases, the usage duration can extend significantly, ranging from several months to up to a year, depending on the stability of the compounded PN or DP.

A key issue lies in the labelling of these IV bags. Technical data sheets often provide limited information, generally specifying their intended use only for short-term applications. Manufacturers typically advertise these bags for nominal PN preparations, indicating suitability for use over a duration of 24–48 h, consistent with standard PN infusion timelines. To address concerns related to long-term use, some manufacturers have developed IV bags with additional polymer layers. These enhancements improve the physical and chemical barrier properties of the bags, ensuring better product integrity during extended contact with compounding. While this added functionality is promising, the details of these advancements are often clarified only through discussions with industrial representatives rather than being explicitly stated in product documentation.^118–122,129^

#### PVC IV bags

PVC IV bags are commonly manufactured with a single-layered structure, selected for the flexibility, transparency, ease of processing and cost-effectiveness. The attached tubes and ports can also be made of PVC or, other materials like polyolefin polymers, for compatibility reasons with the DP. These IV bags are versatile, purposed nowadays for enteral nutrition, rehydration therapy, and the addition of anticoagulants, antibiotics and analgesics. They are also suitable for blood transfusions, dialysis and media culture.

PVC is an amorphous polymer, made of vinyl chloride units. The presence of chlorine in each monomer gives PVC interesting features such as its chemical resistance and rigidity. However, for medical device applications like IV bags, rigidity is not practical. To render the polymer more flexible, a plasticiser is added, which embeds itself in between PVC chains, reducing the intermolecular force between the chains. As a result, this is going to increase the intermolecular space between the polymer chains, thus enabling them to move freely, giving PVC its known flexibility. The concentration of plasticiser required in PVC depends on the application, which in this case would be 30%–50% needed for hospital IV bags. Historically speaking, DEHP was known to be the most commonly used plasticiser in PVC medical products. However, due to toxicity concerns and its role in sorption phenomena, DEHP has been banned in Europe and replaced by safer alternatives such as DEHT, TOTM and others.

That said, due to the required non-negligible concentration of plasticisers required in a PVC, and their potential migration in a DP, less PVC materials are being used for IV therapy due to inherent risk of interactions. While PVC IV bags remain a cost-effective option, hospitals are increasingly adopting safer alternatives like EVA and co-extruded polyolefin, as they offer superior safety profiles, enhanced compatibility with sensitive DPs and reduced risks associated with plasticiser migration, making them a preferred choice for hospital medical device applications.^118–122,128,129,132^

#### EVA IV bags

EVA IV bags are increasingly being adopted by hospitals worldwide, in replacement to PVC. EVA IV bags come in two types structures: monolayer and multilayer. The selection of these layered bags depends on the nature of the compounding. Monolayer EVA IV bags are purposed for hydration, electrolyte solutions, or PN that do not require stringent barrier protection. They are built for short- to medium-term storage, for example, a few weeks, depending on the stability of the product. As for multilayer EVA bags, they are constructed with three layers, including polyamides or polyolefins, for improved barrier properties, durability and chemical resistance, to protect oxygen- or moisture-sensitive formulations for an extended duration of storage, usually up to a year. Tubes and ports attached to EVA IV bags are manufactured from polyolefins, PC, EVA and PVC (the exterior layer), ensuring both strength and compatibility with the drug formulation. EVA is increasingly regarded as a safer alternative to PVC due to its lower extractable profile. EVA’s flexible nature means that it requires fewer plasticisers and is more compatible with lipid- or protein-based drugs compared to PVC.

EVA is a semi-crystalline copolymer, composed of ethylene and vinyl acetate (VA) monomers. This high-performance copolymer possesses very interesting mechanical properties like flexibility, transparency and chemical inertness. The degree of crystallinity and amorphous behaviour in EVA material is influenced by the proportion of VA content. An excessive amount of VA content could make the material rigid while too little could render it too soft. Therefore, a balance of proportion of VA content within the copolymer must be ensured. Therefore, the proportion of VA content in an EVA IV bag should range between 15% and 30%, striking a balance between flexibility and mechanical strength for medical applications.

However, EVA is not autoclavable, unlike PVC, because of its relatively low melting point (80°C–96°C). To address this issue, alternative sterilisation methods, such as gamma irradiation, ethylene oxide or filtration, are recommended, therefore being ideal options for low-temperature sterilisation. If an alternative material is required, polyolefin-based IV bags could be ideal for autoclaving.^118–122,133^

#### Polyolefin IV bags

Polyolefin IV bags have become a popular alternative to PVC and EVA IV bags. Like EVA bags, polyolefin IV bags are available in both monolayer and multilayer versions. Monolayer polyolefin bags are commonly used for short-term applications including IV fluids, for example, saline, dextrose solutions and PN preparations. Multilayer polyolefin bags, like their EVA counterparts, are designed to improve barrier properties and extend the shelf life and stability of DPs, such as biologics, chemotherapy drugs and lipids.

Polyolefin-based IV bags are manufactured using PP, PE or a mix of the two. Both PP and PE are semi-crystalline isotactic polymers that have been rendered flexible for its purpose through the use of advanced manufacturing techniques like co-extrusion, to instil to the polymer strength, elasticity and chemical resistance. These bags are engineered to address concerns such as the leaching of plasticisers from PVC and the limited thermal resistance of EVA.

As for disadvantages, polyolefin bags are known to be costly and even more for multilayered designs due to the complexity of the manufacturing processes. Moreover, polyolefin bags are generally less flexible than EVA IV bags and the reduced flexibility is even more pronounced in multilayered versions, making them particularly inconvenient during mixing of fluids and DPs and infusion. Despite these drawbacks, polyolefin bags remain a highly effective and reliable alternative thanks to their improved stability and protection. Their optimal properties make them ideal primary packaging in the industry for a range of applications.^118–122^

## Expert insights and solutions

### Awareness of the off-label use of primary packaging in hospital pharmacies

In hospital pharmacies, the absence of regulations and viable solutions has resulted in minimal awareness of CCI-related risks among pharmacists. Hospitals should invest in continuing education for pharmacists and compounding technical staff, focusing on the importance of E&L testing, its impact on drug safety and efficacy and especially why primary packaging with high-quality material are essential for this field of work. By increasing awareness of these risks, pharmacists will be better equipped to identify situations in which E&L testing is necessary and take appropriate action to protect patient safety.

International/national conferences and programs/seminars could be organised via state funding or by regulators or industries for hospital pharmacists, healthcare providers, industrials and regulatory bodies to promote and discuss matters related to the use of off-label primary packaging as well as certain case studies to show what should and should not be done. Both industrial and hospital pharmacists could provide a better understanding of both sides of production/practices, a better selection of primary packaging and the implementation of suitable primary packaging adapted for hospital pharmacy compounding.

### Internal guidelines and regulatory reforms

Owing to the safety concerns arising from the off-label use of primary packaging in hospital pharmacy compounding, several regulatory changes are needed, which include the establishment of formal internal guidelines and regulatory reforms specific to off-label uses. New regulations and guidelines should mandate regular risk assessment in hospital pharmacies via E&L analysis across Europe and North America, especially for high-risk compounded DPs, involving off-label use of primary packaging for long-term storage in frail and vulnerable patient populations, all with the aim of ensuring that leachable compound migration does not compromise the safety and stability of these medications over the intended period of use.

Moreover, the lack of clear regulations/guidelines is further complicated by the absence of formal guidance on risk mitigation. Unlike industries that have established analytical and toxicological procedures to assess the risks associated with medical devices and container closure systems for market release, hospital pharmacy compounding lacks such frameworks. It is crucial to ensure that any leachable compounds present are kept to a minimum for frail and vulnerable patients of all ages.

Regulatory bodies, such as the FDA and EMA, as well as local authorities, should stay informed about the latest research and best practices in hospital pharmacy compounding and collaborate with industry experts and hospital pharmacies to develop internal guidelines to regularly assess and update their packaging selection processes. Moreover, should hospitals be required to use an off-label primary packaging for a certain DP; then, any adverse events related to off-label device use in compounding should be reported as post-market surveillance, and regulators should establish clear mechanisms for addressing such reports. Notably, the EU and FDA MDR apply to manufacturers and other economic operators but not to hospitals. The hospital would have to report to the manufacturer (or economic operators), who then have to fulfil their reporting obligations under the MDR.

**Figure 3.**
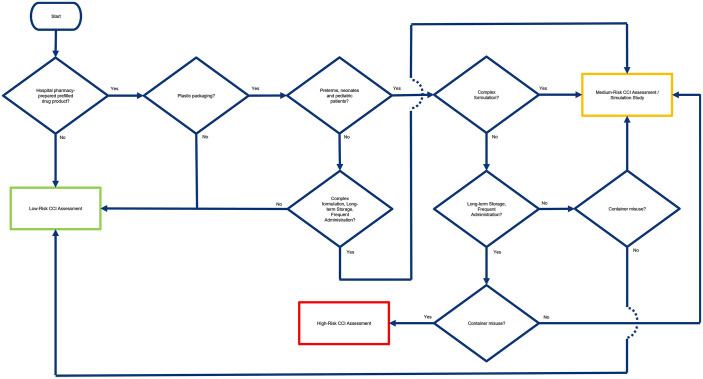
Overall summary of the DP risk assessment qualification workflow in hospital pharmacies. DP, drug product.

Workflows could be proposed in hospital pharmacies for routine risk assessment when facing off-label primary packaging of polymeric nature to standardise the process for hospital pharmacy compounding E&L testing. In these workflows, tasks are performed to help the operator make decisions. One such workflow could be used to qualify DPs conditioned in an off-label medical device for safety risk assessment, allowing the user to determine which compounded DPs are most suited for risk assessment. Importantly, risk is a multiplication of hazard and exposure. The combination of different hazards and exposure factors determines the degree of risk assessment required.^134,135^ The degree of E&L-related risk assessment is based on the type of material, the type of patients, the type of formulations, the frequency of administration, the duration of storage and the original purpose of the applied container. Three types of risk assessments could be proposed: high-risk and medium-risk situations qualify for toxicological assessments, while low-risk situations would not be qualified for further assessment and would be assumed to be safe. Figure 3 describes the risk-determination workflow in hospital pharmacies for qualifying DPs for risk assessment.

It is important to highlight that glass primary packaging, such as vials and syringes, along with secondary components like rubber stoppers and plunger heads, is excluded from this workflow due to their low risk of E&L for hospital patients. These materials, including high-quality borosilicate glass (USP/EP Type 1) and specially coated rubber components, are meticulously manufactured and toxicologically evaluated to ensure suitability for the long-term storage of drug solutions. Hospital pharmacists procure such packaging exclusively from trusted suppliers, adhering to stringent Pharmacopoeia and GMP standards, aligning with industrial best practices for final compounded products. As a result, the focus of E&L concerns is limited to polymer-based primary packaging because of their likeliness of off-label use. However, it is crucial to note that such packaging should not be entirely exempt from E&L testing. Whether compounding is performed using off-label or compliant primary packaging, all compounded preparations should undergo E&L testing to safeguard patient safety and uphold the highest quality standards, especially when batch production is involved.

Hospital pharmacies face ongoing challenges as they wait for the pharmaceutical industry to provide viable solutions that specifically address their unique needs for long-term storage of compounded medications. These solutions must address several critical aspects of E&L risk: primary containers, secondary packaging, labelling and graduation marks. As hospital pharmacies continue to expand their role in compounding and storage, the need for these customised solutions becomes even more critical. Collaboration between the pharmaceutical industry and hospital pharmacy leaders is essential to develop products that meet the unique requirements of hospital compounding and long-term storage.

### Hospital-pharmacy-specific leachable evaluation approach

E&L is an expensive practice, and since legal aspects of hospital pharmacy concerning E&L-related matters are currently non-existent in the eyes of national and international authorities, for example, the FDA and EMA, hospital pharmacies can leverage this situation as an opportunity to adopt a flexible approach in establishing analytics. This should be considered not just on improper use of primary packaging but on all hospital pharmacy batch production, in combination with other factors such as long-term storage, frail and vulnerable patients, frequent administration and various types of formulations. While E&L risk assessments are well established across different categories, including manufacturing, medical devices and pharmaceuticals, there remains a clear need for a fourth category focused solely on hospital pharmacy batch compounding, involving the development and application of an E&L framework. To do so, a balance must be struck: a ‘Goldilocks standard’ via the adopt and adapt approach; that aligns industrial methodologies with hospital realities. Each E&L category is differently approached, with their own scientific logic proven, and in the hospital pharmacy category, it should not be done any different, basing it on hospital pharmacy best practices. Such an approach would promote cost-effective, practical alternatives by taking in more innovative, adaptable solutions rather than applying overly complex, one-size-fits-all industrial practices. Scaling down these methods could be important to allow hospital pharmacies to adopt solutions based off their budgets and compounding needs without compromising safety.

Risk assessment could be performed by larger hospital pharmacy establishments with a quality control or research laboratory with expensive but optimal analytical instruments, such as those used in high-resolution mass spectrometry detection. A hospital-pharmacy-adapted leachable evaluation procedure could be used to perform minimal risk assessments. This system has been tested on multiple hospital-pharmacy-prepared DPs in which leachable compounds were observed, and estimative toxicology assessments were performed.^15,16,136^ The approach for studying plastic-related compounds in hospital-pharmacy-prepared RTA DPs should be based on a series of criteria adapted to current conditions in hospital pharmacies, such as robust, reliable, fast, flexible and operator- and resource-friendly. This system would benefit hospital pharmacies, especially state-run hospitals, where financing and time are always under constraints. Laboratories in hospital pharmacies differ from those in pharmaceutical industries in terms of size, staff and workload. Given their smaller scale and limited workforce, the ability to conduct quick and straightforward analyses would provide a significant advantage. Moreover, hospital pharmacies could benefit from a fast ‘one-shot’ and reliable approach (bypassing extractable studies) that gives the user robust responses and does not require the involvement of E&L-experienced operators because the system would perform all identifications and other necessities efficiently. Different hospital pharmacies could join forces to perform complete analytical and toxicological assessments by combining results for volatile, semivolatile and nonvolatile compounds as well as trace elements. However, these means of mitigating leachable monitoring costs could lead to differences in best practices among hospital pharmacy establishments. Therefore, standardising practices among different laboratories is highly important for standardising responses.

Importantly, industrial E&L remains by far the best practice for material characterisation, plastic-related compound evaluation, RTA DP studies, and toxicological evaluation for safety assessment.^5,20–23,137–139^

### Hospital-pharmacy-adapted medical containers for long-term storage compounding

The other solution would be to use hospital pharmacy-adapted medical containers suitable for specific long-term storage of batch compoundings. Currently, most hospital pharmacies use administration-purposed primary packaging for short- and long-term storage. This choice is often driven by cost and time considerations, despite the outdated nature of this practice. This could come with CCI-related risks to more specific, frail and vulnerable populations since these containers are untested and lack labelling for interactions with DPs. If the industry had the option of marketing medical containers adapted for long-term hospital storage – that is, containers that were already sterilised and packaged individually, such as administration-purposed primary packaging, which were also not dependent on expensive filling equipment – then this market-based approach would not only be a better alternative for hospital pharmacies but also bring about less work in CCI-related risk assessment since the industry would already cover those risks under current regulations. This approach would empower hospital pharmacies to compound DPs for storage durations ranging from a day to over a year, contingent upon stability testing. Moreover, these medical containers could contain E&L low-risk graduation markings and labels that do not require specialised printers.

### Outsourcing

An alternative approach could involve outsourcing these analyses to subject matter experts (SMEs) and private laboratories, along with conducting risk assessments through toxicology consultants. This strategy has the potential to yield higher quality and more precise responses. However, while this approach may be optimal, it could also incur additional costs. Some SMEs would even ask industries for technical information on medical containers or outsource analysis to independent laboratories to establish risk assessments. However, most of the time, this information would turn out to be confidential for proprietary reasons, and subcontracting with industries and private laboratories can be very costly, resulting in a lack of control of the results. All these considerations prompt hospital pharmacy analytics to devise methodologies for monitoring leachable compounds.

The compounding of DPs in hospital pharmacies cannot be halted since small batches of compounded DPs are made to serve patients, ensuring the continuity of the treatment in the most secure, efficient and effective manner. A solution could be to transfer production to a SME or an industry where risk assessment could also be performed. However, the amount of production would not fit the industrial scale due to the small-scale batches needed in hospital pharmacies; therefore, this would be less economically advantageous for the industry. Moreover, overall technological transfer is costly for hospital pharmacies to maintain, which ultimately brings us to our initial answer: compounding must remain in hospital pharmacies.

## Limitations and recommendations

After having discussed about a series of expert opinions on the subject of off-label primary packaging use in hospital pharmacy batch compounding, several potential limitations such as time-consumption, stakeholder resistance and misalignment of solutions can emerge.

### Limitations

The development and implementation of regulatory frameworks, especially mandatory risk assessments, could be a complex and time-consuming process, requiring careful full attention to ensure that hospital pharmacists and other scientific stakeholders understand and adopt the changes. Even once frameworks are in place, hospital pharmacy-specific analytical and toxicological workflows for batch compounding, particularly when off-label primary packaging is involved, demands substantial investment in infrastructure and training, further prolonging resolution. These delays could be further worsened with information gaps, such as the lack of reported cases and limited access to concrete risk data, which could hinder effective communication and stakeholder understanding.

A major hurdle is resistance from stakeholders. Raising awareness among diverse stakeholders, such as healthcare providers, hospital pharmacists, industrial experts and regulators, is no small feat, especially promoting awareness about the risk of using off-label primary packaging and the adoption of a risk-based E&L approach. Some hospital pharmacists and regulators may favour familiar practices and expressing concerns about increased complexity or workload, thus calling it an unrealistic approach. This could lead to a dismissal and may result in finding it all unnecessary regulatory burdens, as they fear disruptions.

E&L analysis can be very expensive for state-funded hospitals, where the technical and financial barriers are often overwhelming. Addressing this challenge would require government support, to encourage the adoption of these practices. However, convincing them to allocate resources to a process traditionally associated with industry may be challenging.

Industrial primary packaging and advanced E&L approaches are frequently too sophisticated to meet the real-world requirements of hospitals, which makes them less practicable in hospital pharmacy environments. Divergent priorities could make collaboration even more difficult: hospitals and regulators prioritise cost-effectiveness and patient safety, while industrial players prioritise profitability and scalability. These conflicting goals may cause problems with trust. Regulators may be sceptical of hospital-driven efforts, doubting their viability or comprehensiveness, while hospitals may perceive industry players as promoting needlessly costly solutions.

### Recommendations to these limitations

Without a doubt, promoting these changes is extremely difficult and challenging, but it is worth in the long run. It is crucial to understand that when patient safety is at stake, hospitals are ethically responsible for their safety, particularly when off-label primary packaging is involved in hospital pharmacy batch production. Industries have already adopted these measures to mitigate risks, and hospitals should ethically follow suit. The stakes are no different.

The organisation and promotion of awareness is extremely challenging, but should not be discouraged. It would require a considerable amount of effort, persistence and patience, which would take a long time to achieve before meaningful results can be achieved. Communication and collaboration are two essential points to maintain, to minimise resistance and facilitate smoother adoption. Implementation should be promoted in different phases in a progressive manner to allow stakeholders enough time to learn and adapt what must be done. Stakeholder priorities should not be neglected, by reducing worries about taking up more duties, and focusing on more practical, cost-effective solutions that would work for everyone involved.

By taking these steps, hospital pharmacies can adopt safer, more reliable practices to safeguard patient safety while meeting the needs of all involved.

## Conclusion

The introduction of centralised RTA DPs has significantly enhanced patient safety in pharmaceutical compounding. This innovation has not only improved the accuracy and consistency of drug formulations but also led to a notable reduction in product waste. These advancements represent crucial steps forwards in optimising hospital pharmacy operations and resource utilisation.

A brief examination of the historical development of medical devices used in hospital wards reveals the intricate relationship between industries and hospital pharmacies. This evolution has been characterised by continuous improvements in design, materials and functionality, all aimed at enhancing patient care and safety. Production by hospital pharmacies remains of utmost importance for the continuity of patient treatment and safety. However, several risk factors must be carefully managed in hospital pharmacy production, such as the improper use of primary packaging, due to the lack of awareness, financial constraints and/or inadequate regulations, along with patient-specific factors, storage duration, administration frequency and formulation type, which could lead to the CCI-related issues including migration of potentially harmful leachable compounds and container–content interactions, resulting in further patient safety concerns.

A thorough, multidisciplinary and multiprofessional approach to risk assessment is essential to ensure the highest standard of patient safety and treatment effectiveness. This approach includes conducting regular and comprehensive risk evaluations, implementing robust quality control measures, providing ongoing training for pharmacy staff, remaining informed of the latest research, proposing regulatory guidelines and fostering open communication among all stakeholders in the medication use process.

By meticulously addressing these aspects, hospital pharmacies can significantly increase the safety and effectiveness of their production processes, ultimately contributing to improved patient outcomes and healthcare quality.

## Declarations

**Ethics approval and consent to participate:** Not applicable.

**Consent for publication:** Not applicable.

**Availability of data and materials:** Not applicable since it is a review.
